# Lipopolysaccharide Preparation Derived From *Porphyromonas gingivalis* Induces a Weaker Immuno-Inflammatory Response in BV-2 Microglial Cells Than *Escherichia coli* by Differentially Activating TLR2/4-Mediated NF-κB/STAT3 Signaling Pathways

**DOI:** 10.3389/fcimb.2021.606986

**Published:** 2021-03-18

**Authors:** Che Qiu, Zhen Yuan, Zhiyan He, Huiwen Chen, Yue Liao, Shiliang Li, Wei Zhou, Zhongchen Song

**Affiliations:** ^1^ Department of Periodontology, Shanghai Ninth People’s Hospital, College of Stomatology, Shanghai Jiao Tong University School of Medicine, Shanghai, China; ^2^ National Clinical Research Center for Oral Diseases, Shanghai Key Laboratory of Stomatology and Shanghai Research Institute of Stomatology, Shanghai, China; ^3^ Shanghai Key Laboratory of New Drug Design, State Key Laboratory of Bioreactor Engineering, School of Pharmacy, East China University of Science and Technology, Shanghai, China; ^4^ Laboratory of Oral Microbiota and Systemic Diseases, Shanghai Ninth People’s Hospital, College of Stomatology, Shanghai Jiao Tong University School of Medicine, Shanghai, China

**Keywords:** *Porphyromonas gingivalis*, lipopolysaccharide, BV-2 microglial cells, immuno-inflammatory, TLRs, NF-κB signaling, STAT3 signaling, molecular docking

## Abstract

Alzheimer’s disease (AD) is a degenerative disease of the central nervous system with unclear etiology and pathogenesis. In recent years, as the infectious theory and endotoxin hypothesis of AD has gained substantial attention, several studies have proposed that *Porphyromonas gingivalis* (*P. gingivalis*), one of the main pathogenic bacteria of chronic periodontitis, and the lipopolysaccharide (LPS) of *P. gingivalis* may lead to AD-like pathological changes and cognition impairment. However, research on the relationship between *P. gingivalis*-LPS and neuroinflammation is still lacking. Our study aimed to investigate the effects of *P. gingivalis*-LPS preparation on immuno-inflammation in microglial cells and further compared the differential inflammatory response induced by *P. gingivalis*-LPS and *Escherichia coli* (*E. coli)* LPS preparations. The results showed that *P. gingivalis*-LPS could upregulate the gene expression and release of pro-inflammatory factors in BV-2 microglial cells, including IL-1β, IL-6, TNF-α, IL-17, and IL-23. We also observed an increase in the level of Toll-like receptor 2/4 (TLR2/4) and NF-κB/STAT3 signaling. Moreover, the changes mentioned above were more significant in the *E. coli*-LPS group and the effects of both kinds of LPS could be differentially reversed by the administration of the TLR2 inhibitor C29 and TLR4 inhibitor TAK-242. The molecular simulation showed that the binding affinity of *P. gingivalis*-lipid A to TLR4-MD-2 was weaker than *E. coli*-lipid A, which was probably due to the presence of fewer acyl chains and phosphate groups of *P. gingivalis*-lipid A than *E. coli*-lipid A. We conclude that *P. gingivalis*-LPS could activate TLR2/4-mediated NF-κB/STAT3 signaling pathways, which ultimately resulted in an immune-inflammatory response in BV-2 microglia. In contrast to *E. coli*-LPS, *P. gingivalis*-LPS is a weaker TLR2/4 agonist and NF-κB/STAT3 signaling activator. Furthermore, the different fatty acid chains and phosphate groups between *P. gingivalis*-lipid A and *E. coli*-lipid A may be the reason for the weaker activating properties of *P. gingivalis*-LPS.

## Introduction

Alzheimer’s disease (AD) is a degenerative disorder of the central nervous system (CNS) characterized by progressive cognitive dysfunction, memory impairment, and other dementia manifestations, and it has been identified as the most common cause for dementia (Abeysinghe et al., 2020). To date, the etiology and pathogenesis of AD are still unclear, but several hypotheses have been proposed to explain the AD pathological mechanisms, including the amyloid theory, tau protein theory, and neuroinflammation theory (Chen, 2018).

Drugs currently approved by the American Food and Drug Administration for AD generally target the symptoms rather than the underlying etiology or pathogenesis, which cannot prevent or reverse the disease progression of AD (Gao et al., 2016). In recent years, the infectious theory of AD has gained substantial attention, and some microorganisms, such as *Helicobacter pylori* (Doulberis et al., 2018), *Herpes virus* (Itzhaki, 2017), and gut microbiota, (He et al., 2020) have been reported to be associated with the progression of AD. This suggests a role for infectious agents, and anti-pathogenic microorganism drugs for AD patients may be a possible direction for future drug development (He et al., 2020).

Chronic periodontitis (CP) is one of the most common oral chronic inflammatory and infectious diseases (Papapanou et al., 2018). The infectious theory believes that periodontal pathogens and their virulence factors may invade the CNS by destroying the blood-brain barrier (BBB). Then, once microglia are activated, they induce microglia mutual activation with inflammatory factors and mediate the interaction between neurons and microglia. The resulting Aβ deposition leads to the loss of neuronal function (Wu and Nakanishi, 2017). As one of the most common and well-documented Gram-negative anaerobic pathogens in CP, *Porphyromonas gingivalis* (*P. gingivalis*) has been reported to be highly correlated with AD (Beydoun et al., 2020), and the lipopolysaccharide (LPS) located in the outer membrane of Gram-negative bacteria is a main virulence factor of *P. gingivalis* (Kamer et al., 2020). Recently, *P. gingivalis*-LPS and *P. gingivalis* DNA have been detected in human brain tissue and cerebrospinal fluid of AD patients, respectively (Poole et al., 2013; Dominy et al., 2019). Furthermore, our previous study also indicated that periodontitis induced by topical application of *P. gingivalis*-LPS into the gingival sulcus could contribute to learning and memory impairment *via* neuroinflammation in Sprague-Dawley rats, and the activation of microglia is closely related to disease progression (Hu et al., 2020). These findings indicate that *P. gingivalis* and its virulence factors may infect the CNS, cause neuroinflammation, and eventually AD-like pathological changes (Halliday et al., 2016; Singhrao et al., 2017; Zhang et al., 2018).

As the first line of defense in the CNS and initiate immune responses to injuries and pathogens, microglia play a vital role in the pathological process of AD, while LPS is a strong stimulator of microglial activation. Abnormally activated microglia can significantly accelerate neuroinflammatory and neurotoxic responses by releasing various proinflammatory cytokines and mediators (Kirkley et al., 2017; Hickman et al., 2018), and neuroinflammation will eventually lead to synaptic degeneration, neuronal cell death, and cognitive dysfunction (Dansokho and Heneka, 2018). However, in most AD-related studies, the immune-inflammation of microglia cells was induced by LPS derived from *E. coli*, which is also Gram-negative bacteria like *P. gingivalis* (Subedi et al., 2017; Xu et al., 2018). *E. coli*-LPS can affect NF-κB, STAT3, and/or other transcription factors in the nucleus by activating TLR4 and may trigger the release of proinflammatory cytokines (Hines et al., 2013; Lester and Li, 2014; Fellner et al., 2017; Nam et al., 2018). *P. gingivalis*-LPS and *E. coli*-LPS have different molecular structures (Ogawa et al., 2007), and both types of LPS can differentially modulate the expression of Toll-like receptors (TLRs) and inflammatory cytokine responses in certain cell lines (Martin et al., 2001; Nakamura et al., 2008; Jones et al., 2010), while the effect of *P. gingivalis*-LPS on microglial cells is still unclear.

Our study aimed to investigate the effect of *P. gingivalis*-LPS preparation on immuno-inflammation in microglial cells and further compared the differential inflammatory response induced by *P. gingivalis*-LPS and *E. coli*-LPS preparations.

## Materials and Methods

### Antibodies and Inhibitors

The following primary and secondary antibodies were used for western blotting (WB) or Immunofluorescence (IF) assays: rabbit anti-TLR2 (1:1000 for WB, Absin Bioscience, Shanghai, China), rabbit anti-TLR4 (1:1000 for WB, Absin Bioscience), rabbit anti-CD14 (1:1000 for WB, Absin Bioscience), rabbit anti-phosphorylated (p)-p65 (1:1000 for WB, Cell Signaling Technology, Boston, Massachusetts, USA), rabbit anti-p65 (1:1000 for WB, Cell Signaling Technology), rabbit anti-p-STAT3 (Tyr705, 1:1000 for WB, Cell Signaling Technology), rabbit anti-STAT3 (1:1000 for WB, Cell Signaling Technology), HRP-labeled goat anti-rabbit IgG (1:1000 for WB, Beyotime Biotechnology, Shanghai, China), rabbit anti-p65 (1:100 for IF, Beyotime Biotechnology), rabbit anti-p-STAT3 (Tyr705, 1:100 for IF, Absin Bioscience), and Cy3-labeled goat anti-rabbit IgG (1:1000 for IF, Beyotime Biotechnology).

We used a TLR4 inhibitor (TAK-242, 1μM, MedChemExpress, Monmouth Junction, NJ, USA) and a TLR2 inhibitor (C29, 100μM, MedChemExpress) in our experiments. LPS-Standard derived from *P. gingivalis* or *E. coli* 055: B5 was purchased from InvivoGen (San Diego, CA, USA).

### Cell Line and Culture Condition

The BV-2 microglia cell line was purchased from the Cell Resource Center, Institute of Basic Medical Sciences Chinese Academy of Medical Sciences (Beijing, China). BV-2 microglial cells were maintained in DMEM (Thermo Fisher Scientific, Waltham, MA, USA) with 5% fetal bovine serum (FBS, Thermo Fisher Scientific), 1% penicillin-streptomycin (100μg/mL, Thermo Fisher Scientific), and 1% GlutaMAX™-I (100μg/mL, Thermo Fisher Scientific) at 37°C with 5% CO_2_ and sub-cultured every 3 or 4 days. Cells were re-plated on 6- or 96-well Cell Culture Microplates (2×10^5^ cells/well in 6-well microplates and 5×10^3^ cells/well in 96-well microplates; Corning Life Sciences, NY, USA). After the culture medium was replaced with fresh FBS-free medium, BV-2 microglial cells were pre-treated with TAK-242 (1μM) or C29 (100μM) for 60min, then LPS (1μg/mL) was added to the culture medium and treated for different times.

### Reverse-Transcription and Real-Time PCR

After LPS simulation, total RNA was extracted from BV-2 microglial cells with E.Z.N.A.^®^ Total RNA Kit I (Omega Bio-tek, Georgia, USA) according to the manufacturer’s instructions. A total of 1000ng of extracted RNA was reverse-transcribed to cDNA using a PrimeScript™ RT reagent Kit (Takara, Otsu, Shiga, Japan). The primer sequences specific to signaling pathways (TLR2, TLR4, CD14, NF-κB p65, STAT3, and GAPDH) and Inflammatory cytokines (IL-1β, IL-6, TNF-α, IL-17A, IL-23, and β-actin) for BV-2 microglial cells are shown in **Table 1**. Real-time PCR was performed in a LightCycler480 system (Roche, Basel, Switzerland) using TB Green^®^ Premix Ex Taq™ (Takara, Otsu, Shiga, Japan). The DNA amplification was performed as follows: the first cycle was maintained at 95°C for 30s followed by 40 cycles consisting of denaturation (95°C for 10s), annealing, and extension (60°C for 30s). The values obtained for the target gene expression were normalized to β-actin and quantified relative to the expression in control samples using the 2^−ΔΔCt^ method.

**Table 1:** The primer sequences for BV-2 microglial cells.

Target Gene	Primer Sequences (5’ to 3’)
TLR2	(F) GCT CCT GCG AAC TCC TAT CC (R) CAG CAG ACT CCA GAC ACC AG
TLR4	(F) CGC TTT CAC CTC TGC CTT CAC TAC AG (R) ACA CTA CCA CAA TAA CCT TCC GGC TC
CD14	(F) TGT CGT GGG CAA CAA GGG ATG (R) AAG GTG GAG AGG GCA GGG AAG A
NF-κB p65	(F) TCG AGT CTC CAT GCA GCT ACG G (R) CGG TGG CGA TCA TCT GTG TCT G
STAT3	(F) CCC CCG TAC CTG AAG ACC AAG (R) TCC TCA CAT GGG GGA GGT AG
GAPDH	(F) ACC CAG AAG ACT GTG GAT GG (R) CAC ATT GGG GGT AGG AAC AC
IL-1β	(F) AAA TCT CGC AGC AGC ACA TCA A (R) CCA CGG GAA AGA CAC AGG TAG C
IL-6	(F) ATC CAG TTG CCT TCT TGG GAC TGA (R) TAA GCC TCC GAC TTG TGA AGT GGT
TNF-α	(F) GAC AAG GCT GCC CCG ACT ACG (R) CTT GGG GCA GGG GCT CTT GAC
IL-17A	(F) TGA TGC TGT TGC TGC TGC TGA G (R) CAC ATT CTG GAG GAA GTC CTT GGC
IL-23	(F) GAC TCA GCC AAC TCC TCC AGC CAG (R) TTG GCA CTA AGG GCT CAG TCA GA
β-actin	(F) CAT CCG TAA AGA CCT CTA TGC CAA C (R) ATG GAG CCA CCG ATC CAC A

### Enzyme-Linked Immunosorbent Assay (ELISA)

To explore the effects of *P. gingivalis*-LPS on IL-1β, IL-6, TNF-α, IL-17A, and IL-23 levels in microglial cells in comparison with the effects of *E. coli*-LPS, an enzyme-linked immunosorbent assay (ELISA) was performed. Briefly, BV-2 microglial cells were pre-treated with TAK-242 (1μM) or C29 (100μM) for 60min, then treated with LPS (1μg/mL) for different times. ELISA was used to measure levels of pro-IL-1β, IL-6, TNF-α, IL-17A, and IL-23 using the conditioned medium (IL-6, TNF-α, IL-17A, and IL-23) or cell homogenate (pro-IL-1β). Mouse IL-1β, IL-6, TNF-α, and IL-23 ELISA kits (NeoBioscience, Shenzhen, China) and Mouse IL-17A ELISA kits (Beyotime Biotechnology) were used according to the manufacturer’s recommendations. The absorbance of the samples was measured at 450 nm using a microplate reader (Epoch2, Bio-Tek, Winooski, VT, USA).

### Western Blotting

To explore *P. gingivalis*-LPS effects on TLR2, TLR4, and CD14 expression and NF-κB/STAT3 signaling in BV-2 microglial cells, and further compare these effects with those induced by *E. coli*-LPS, BV-2 microglial cells were treated with TAK-242 (1μM) or C29 (100μM) for 60min, followed by LPS (1μg/mL) stimulation. After the final incubation, the cells were lysed with RIPA buffer containing 2% protease and phosphatase inhibitor cocktail (50mM, Beyotime Biotechnology) and 1% PMSF (100mM, Beyotime Biotechnology). Following the addition of sodium dodecyl sulfate (SDS) loading buffer, the samples were boiled for 5min, and proteins were subsequently detected by western blotting analysis. The proteins were transferred to a polyvinylidene difluoride (PVDF) membrane after separation. A wide range of protein markers was run in parallel to detect the molecular weight of proteins. A 5% skimmed milk solution was used for membrane blockage to reduce nonspecific binding. Proteins were probed with specific antibodies and images were quantified using ImageJ 1.52a software (National Instituted of Health, Bethesda, MD, USA).

### Immunofluorescence Staining

To investigate *P. gingivalis*-LPS and *E. coli*-LPS activation of NF-κB/STAT3 signaling, BV-2 microglial cells were treated with 1μg/mL *P. gingivalis*-LPS or *E. coli*-LPS for different times. After the final incubation, the cells were fixed with 4% paraformaldehyde for 15min. After washing, cells were blocked for 1h at room temperature with blocking solution (3% bovine serum albumin and 0.1% glycine in PBS). They were then incubated with specific primary antibodies for IF overnight at 4°C. The next day, the cells were washed with PBS three times and incubated with fluorescent-labeled secondary antibodies for 1h at room temperature. After washing, the cells were mounted in a DAPI-containing solution (Beyotime Biotechnology). Finally, images were captured using an Inverted Fluorescence Microscope (Leica, Tokyo, Japan) and quantified using ImageJ 1.52a software (National Instituted of Health, USA).

### Statistical Analysis

Data are presented as mean ± standard deviation (SD). The statistical analyses were performed using one-way ANOVA, two-way ANOVA, or Student’s *t*-test with GraphPad Prism 7 software (GraphPad Software, San Diego, CA, USA). Post-hoc analyses were performed with Tukey’s multiple comparison test with significance set at *p<0.05, **p<0.01, and ***p<0.001.

### Computational Methods

The binding mode and affinity between ligands and receptors were obtained using the molecular docking Autodock Vina screening tool (Trott and Olson, 2010), which is a free open-source package with improved speed and accuracy for docking studies. Molecular docking involves three steps: (i) target protein preparation, (ii) ligand preparation, and (iii) and molecular docking.

#### Protein Preparation

Crystal structure of mouse TLR4/MD-2/LPS complex was downloaded from Protein Data Bank (PDB ID: 3VQ2). AutodockTools (ADT version 1.5.6) was used for protein optimization by removing water, ligand, and other heteroatoms. The missing hydrogen atoms of the protein were added, and then the non-polar ones were merged. Gasteiger charges were calculated. The prepared protein was saved as a PDBQT file.

#### Ligand Preparation

The structure of LPS consists of lipid A, core oligosaccharide, and O-antigen. Lipid A is the amphipathic glycolipid moiety of LPS. It stimulates the immune system by tightly binding to the TLR4-MD2 receptor (Hashimoto et al., 2003). *E. coli*-lipid A was derived from the crystal structure (PDB ID: 3VQ2), and then hydrogens were added in Maestro (Schrödinger Inc., version 11.1). The Epik module in Maestro was used to quickly and accurately predict the pKa of *E. coli*-lipid A, while the 3-dimensional (3D) structure of *E. coli*-lipid A was minimized using the OPLS3 forced field. Since the four reported *P. gingivalis*-lipid A forms (5*P. g*-lipid A, 4a*P. g*-lipid A, 4b*P. g*-lipid A, and 3*P. g*-lipid A) were similar to the structure of *E. coli*-lipid A, their 3D structures were built based on the 3D structure of *E. coli*-lipid A using the Build module in Maestro. Then the 3D structures of lipid A were individually saved as a mol2 file.

**Figure 1:**
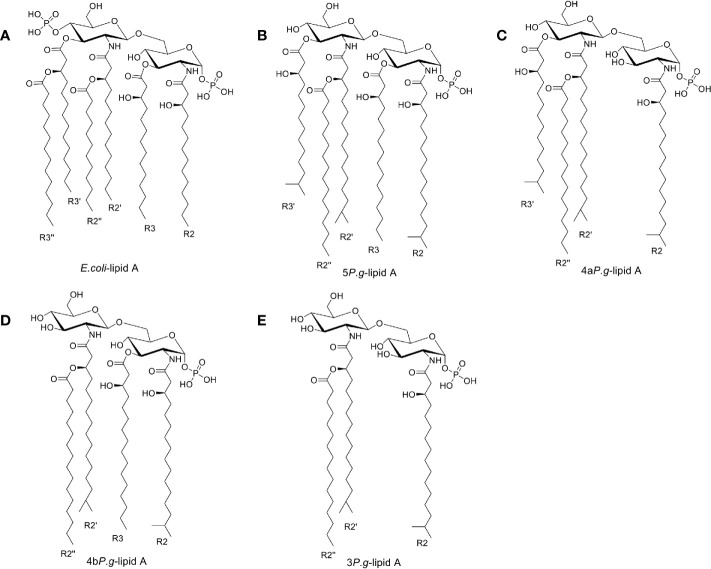
Chemical structures of Lipid A. **(A)** Lipid A structure found in *E. coli*-LPS. **(B**–**E)**. Three lipid A species found in the LPS preparation of *P. gingivalis* (Dixon and Darveau, 2005) containing 5, 4, or 3 fatty acids, named as 5*P. g*-lipid A, 4a*P. g*-lipid A, 4b*P. g*-lipid A, and 3*P. g*-lipid A, respectively. The majority of bacterial lipid A structures are conserved and serve as the hydrophobic anchor of LPS on the majority of bacterial outer membranes.

The above five mol2 files were then imported into AutodockTools to calculate the Gasteiger charges, determine the root of the ligands, and set the flexible bonds. There are >50 flexible bonds in the lipid A structures, and we set a part of the molecule as rigid in order to avoid obtaining non-converged solutions (**Figure 1**). The prepared structures were saved as separate PDBQT files.

#### Molecular Docking

Depending on where the protein and crystalized ligand interacted, the grid size was set to 76×48×64 Å^3^, and the grid center was designated at dimensions (x, y, and z) of -28.8, -15.15, and 22.8. Autodock Vina was employed for docking with gird box properties in the configuration file. The maximum number of binding modes generated was set to 200. These parameters were applied to all lipid As. PyMOL (version 2.3.0) was used to obtain the pdb complex file.

## Results

### Effects of LPS on the Expression of TLRs in BV-2 Microglial Cells

**Figure 2:**
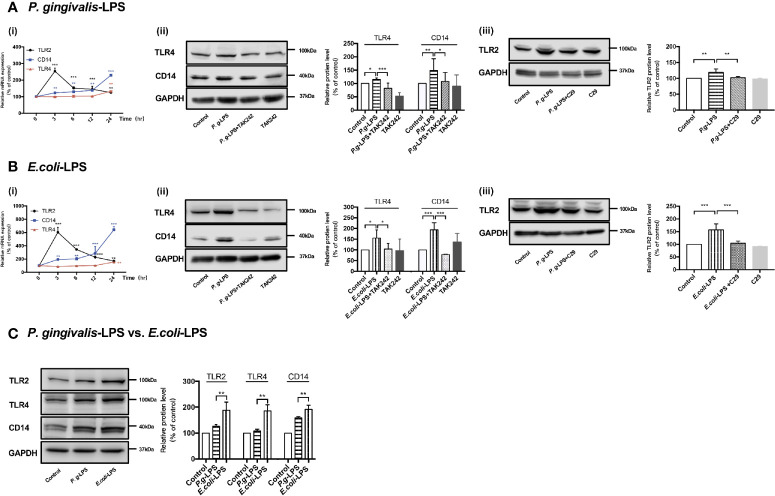
Effects of *P. gingivalis*-LPS and *E. coli*-LPS on gene and protein expression of TLR2, TLR4, and CD14 in BV−2 microglial cells. (Ai, Bi) BV-2 microglial cells were treated with 1μg/mL *P. gingivalis*-LPS or *E. coli*-LPS for different times, and RT-PCR was performed; two-way ANOVA, **p < 0.01, and ***p < 0.001 compared to the 0hr group. **(Aii, Bii)** BV-2 microglial cells were treated with TAK-242 (1μM) or serum-free medium for 60min, followed by treatment with *P. gingivalis*-LPS, *E. coli*-LPS (1μg/mL) or serum-free medium for 24h and western blotting with anti-TLR4, anti-CD14, and anti-GAPDH antibodies; one-way ANOVA, *p < 0.05, **p < 0.01, and ***p < 0.001 compared to the control group or the LPS groups. **(Aiii, Biii)** BV-2 microglial cells were treated with C29 (100μM) or serum-free medium for 60 min, followed by treatment with *P. gingivalis*-LPS, *E. coli*-LPS (1μg/mL) or serum-free medium for 24h and western blotting with anti-TLR2 and anti-GAPDH antibodies; one-way ANOVA, **p < 0.01 and ***p < 0.001 compared to the control group or the LPS groups. **(C)** BV-2 microglial cells were treated with 1μg/mL *P. gingivalis*-LPS, *E. coli*-LPS (1μg/mL) or serum-free medium for 24h and western blotting with anti-TLR2, anti-TLR4, anti-CD14, and anti-GAPDH antibodies; Student’s *t*-test, **p < 0.01 compared the *P. gingivalis*-LPS group with the *E. coli*-LPS group. Data from three independent experiments are presented as mean ± SD.

As shown in **Figure 2Ai**, RT-PCR assays were performed for TLR2, TLR4, and CD14 genes. Upregulation of TLR2, TLR4, and CD14 mRNA was observed in cells stimulated with 1μg/mL *P. gingivalis*-LPS. Next, WB analysis was performed for TLR2, TLR4, and CD14 protein to investigate the effects of *P. gingivalis*-LPS in BV-2 cells. After 24h of LPS stimulation, the *P. gingivalis*-LPS group induced an approximately 1.2-fold increase in TLR4 protein expression and an approximately 1.5-fold increase in CD14 protein expression in comparison to the control group, respectively. The *P. gingivalis*-LPS plus TAK-242 group showed reduced TLR4 and CD14 protein expression in comparison to the *P. gingivalis*-LPS group (**Figure 2Aii**). Furthermore, The *P. gingivalis*-LPS group induced an approximately 1.2-fold increase in TLR2 protein expression in comparison to the control group, and the *P. gingivalis*-LPS plus C29 group showed reduced TLR2 protein expression in comparison to the *P. gingivalis*-LPS group (**Figure 2Aiii**).

Moreover, as shown in **Figure 2Bi**, upregulation of TLR2, TLR4, and CD14 mRNA was also observed in cells induced by 1μg/mL *E. coli*-LPS, and changes of protein levels induced by *E. coli*-LPS could also be restored by TAK-242 or C29 administration (**Figure 2Bii-iii**). However, differently from *P. gingivalis*-LPS, the *E. coli*-LPS group showed an approximately 1.5-, 2-, or 1.6-fold increase in TLR4, CD14, or TLR2 protein expression in comparison to the control group, respectively (**Figure 2Bii-iii**), and significant differences were observed between the *P. gingivalis*-LPS group and *E. coli*-LPS group in TLR4, CD14, or TLR2 protein expression (**Figure 2C**), which demonstrated the abilities of these two kinds of LPS preparations modulate the expression of members of the TLRs family differently in BV-2 microglial cells.

### Gene Expression and Release of Inflammatory Cytokines in LPS-Stimulated BV−2 Microglial Cells

**Figure 3:**
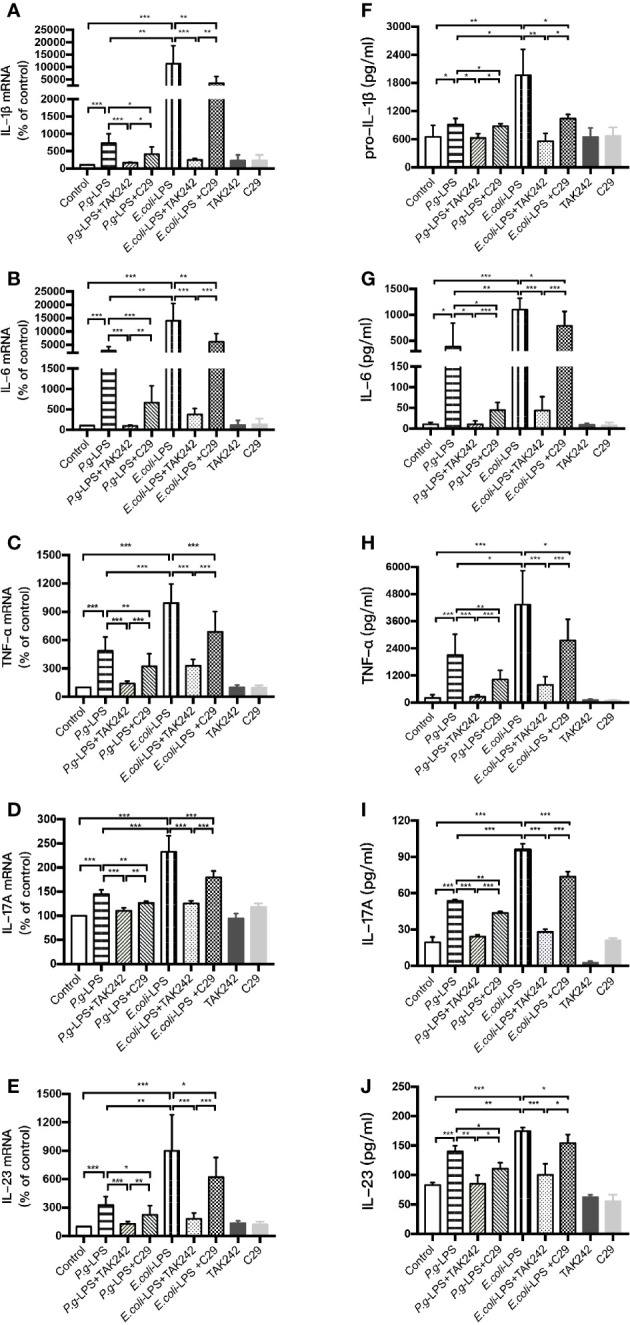
Inflammatory cytokine gene and protein expression in *P. gingivalis*-LPS and *E. coli*-LPS stimulated BV−2 microglial cells. **(A–E)** BV-2 microglial cells were treated with TAK-242 (1μM), C29 (100μM), or serum-free medium for 60 min, followed by treatment with *P. gingivalis*-LPS, *E. coli*-LPS (1μg/mL) or serum-free medium for 6h (IL-1β, IL-6, and TNF-α), 12h (IL-23) or 24h (IL-17A), and RT-PCR was performed. **(F–J)** BV-2 microglial cells were treated with TAK-242 (1μM), C29 (100μM) or serum-free medium for 60min, following by treatment with *P. gingivalis*-LPS, *E. coli*-LPS (1μg/mL) or serum-free medium for 6h (pro-IL-1β, IL-6, and TNF-α), 12h (IL-23) or 24h (IL-17A), and pro-IL-1β, IL-6, TNF-α, IL-17A, and IL-23 levels were measured using ELISA kits. Data from three independent experiments are presented as mean ± SD; Student’s *t*-test, *p < 0.05, **p < 0.01, and ***p < 0.001 compared the *P. gingivalis*-LPS group with the *E. coli*-LPS group; one-way ANOVA, *p < 0.05, **p < 0.01, and ***p < 0.001 compared to the control group, the LPS groups, or the LPS plus TAK-242 groups.

We analyzed the LPS-induced expression of inflammatory cytokine genes in BV-2 cells by RT-PCR and the resulting protein levels were detected by ELISA. After 6h (IL-1β, IL-6, and TNF-α), 12h (IL-23), or 24h (IL-17A) of 1μg/mL LPS simulation, the *P. gingivalis*-LPS group showed an approximately 7-, 30-, 5-, 1.5-, or 3-fold increase in IL-1β, IL-6, TNF-α, IL-17A, or IL-23 gene upregulation in comparison with the control group, respectively (**Figures 3A–E**). Meanwhile, the expression of pro-IL-1β, IL-6, TNF-α, IL-17A, and IL-23 protein in the *P. gingivalis*-LPS group was significantly higher than that in the control group (**Figures 3F–J**). In contrast, the *E. coli*-LPS group showed an approximately 110-, 140-, 10-, 2.5-, or 9-fold increase in IL-1β, IL-6, TNF-α, IL-17A, or IL-23 gene upregulation compared with the control group, and the expression of pro-IL-1β, IL-6, TNF-α, IL-17A, and IL-23 protein in the *E. coli*-LPS group was significantly higher than that of the control group. Significant differences were observed between the *P. gingivalis*-LPS group and *E. coli*-LPS group as mentioned above (**Figures 3A–J**).

Furthermore, the LPS plus TAK-242 groups and LPS plus C29 groups showed decreasing IL-1β, IL-6, TNF-α, IL-17A, and IL-23 mRNA and protein expression in comparison to the LPS group. Compared with plus C29 groups, plus TAK-242 groups were more effective in reducing mRNA and protein expressions (**Figures 3A–J**).

These findings indicated that *P. gingivalis*-LPS could upregulate the level of inflammation cytokine in BV-2 microglial cells but similar up-regulation was weaker than that of *E. coli*-LPS. The effects of LPS were significantly prevented by TLR4 inhibitor TAK-242 or TLR2 inhibitor C29. As the main component of LPS preparations, LPS-Standard derived from *P. gingivalis* and *E. coli* mainly activates TLR4 instead of TLR2, so TLR4 inhibitor was more effective in downregulating inflammatory factors.

### Effects of LPS on the NF−κB Signaling Pathway in BV−2 Microglial Cells

The nuclear transcription factor NF−κB is known to play a critical role in LPS-mediated activation in various cell types. Therefore, activation and phosphorylation of NF-κB p65 were examined with BV-2 cells treated by *P. gingivalis*-LPS and *E. coli*-LPS.

**Figure 4:**
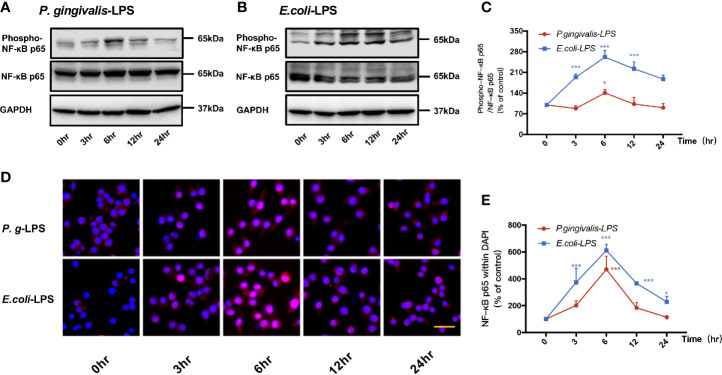
Phosphorylation and nuclear translocation of NF-κB p65 in *P. gingivalis*-LPS and *E. coli*-LPS simulated BV−2 microglial cells. **(A, B)** BV-2 microglial cells were treated with 1μg/mL *P. gingivalis*-LPS or *E. coli*-LPS for different times and western blotting with anti-phosphorylated (p)-NF-κB p65, anti-NF-κB p65, and anti-GAPDH antibodies. **(C)** Semiquantitative analysis of the protein levels. **(D)** BV-2 microglial cells were treated with 1μg/mL *P. gingivalis*-LPS or *E. coli*-LPS for different times, followed by immunofluorescence staining with anti-NF-κB p65 antibody and DAPI. NF-κB p65 and nucleus are colored red and blue, respectively (Microscope images were at a magnification of 100×, Bar=50μm). **(E)** Semiquantitative analysis of the NF-κB p65 protein fluorescence intensity within DAPI. Data from three independent experiments are presented as mean ± SD; two-way ANOVA, *p < 0.05 and ***p < 0.001 compared to the the 0hr group.

**Figure 5:**
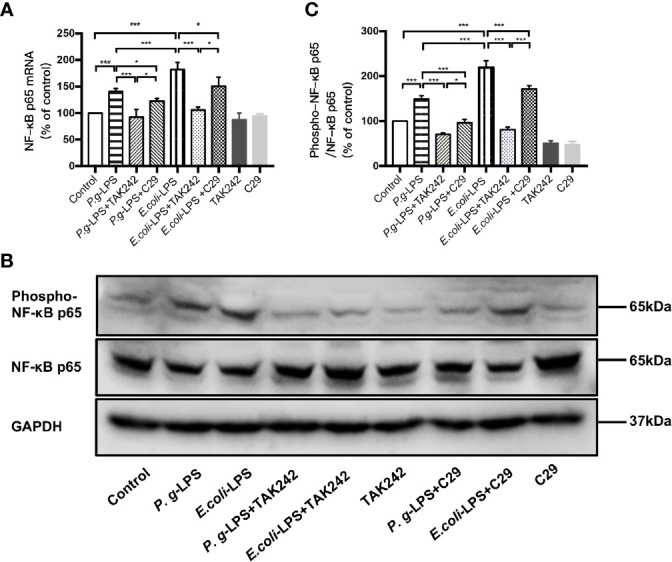
Different effects of *P. gingivalis*-LPS and *E. coli*-LPS on the gene, protein expression, and phosphorylation of NF−κB p65 in BV−2 microglial cells. **(A)** BV-2 microglial cells were treated with TAK-242 (1μM), C29 (100μM), or serum-free medium for 60min, followed by treatment with *P. gingivalis*-LPS, *E. coli*-LPS (1μg/mL), or serum-free medium for 6h, and RT-PCR was performed. **(B)** BV-2 microglial cells were treated with TAK-242 (1μM), C29 (100μM), or serum-free medium for 60min, followed by treatment with *P. gingivalis*-LPS, *E. coli*-LPS (1μg/mL), or serum-free medium for 6h and western blotting with anti-phosphorylated (p)-NF-κB p65, anti-NF-κB p65, and anti-GAPDH antibodies. **(C)** Semiquantitative analysis of the protein levels. Data from three independent experiments are presented as mean ± SD; Student’s *t*-test, ***p < 0.001 compared the *P. gingivalis*-LPS group with the *E. coli*-LPS group; one-way ANOVA, *p < 0.05 and ***p < 0.001 compared to the control group, the LPS groups or the LPS plus TAK-242 groups.

As shown in **Figures 4A, C**, p-p65/p65 protein expression increased within 6h after *P. gingivalis*-LPS stimulation and peaked at 6h. To verify these findings, we performed IF staining with an anti-p-p65 antibody and found that significant nuclear translocation occurred at 6h after stimulation with *P. gingivalis*-LPS and the translocation peaked at 6h (**Figures 4D, E**). Moreover, the elevated expression of NF−κB p65 mRNA and phosphor-p65/p65 protein induced by *P. gingivalis*-LPS was attenuated by the TLR4 inhibitor TAK-242 or TLR2 inhibitor C29 at 6h, and, compared with the C29-treated groups, TAK-242-treated groups were more effective in reducing NF−κB p65 mRNA and p-p65/p65 protein expression (**Figures 5A–C**).

In comparison to *P. gingivalis*-LPS, the *E. coli*-LPS group showed an earlier upregulation of p-p65/p65 protein expression and nuclear translocation of p-p65 (**Figures 4B–E**). Meanwhile, significant differences of NF−κB p65 mRNA and phosphor-p65/p65 protein expression were observed between the *P. gingivalis*-LPS group and *E. coli*-LPS group at 6h (**Figures 5A–C**).

### Effects of LPS on the STAT3 Signaling Pathway in BV−2 Microglial Cells

**Figure 6:**
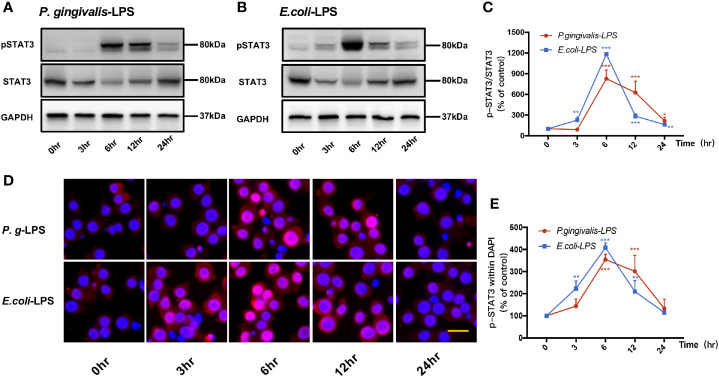
Phosphorylation and nuclear translocation of STAT3 in *P. gingivalis*-LPS and *E. coli*-LPS simulated BV−2 microglial cells. **(A, B)** BV-2 microglial cells were treated with 1μg/mL *P. gingivalis*-LPS or *E. coli*-LPS for different times and western blotting with anti-phosphor-STAT3, anti-STAT3, and anti-GAPDH antibodies. **(C)** Semiquantitative analysis of the protein levels. **(D)** BV-2 microglial cells were treated with 1μg/mL *P. gingivalis*-LPS or *E. coli*-LPS for different times, followed by immunofluorescence staining with anti-phosphorylated (p)-STAT3 antibody and DAPI. P-STAT3 and nuclei are colored red and blue, respectively (Microscope images were at a magnification of 200×, Bar=25μm). **(E)** Semiquantitative analysis of the p-STAT3 protein fluorescence intensity within DAPI. Data from three independent experiments are presented as mean ± SD; two-way ANOVA, *p < 0.05, **p < 0.01, and ***p < 0.001 compared to the the 0hr group.

**Figure 7:**
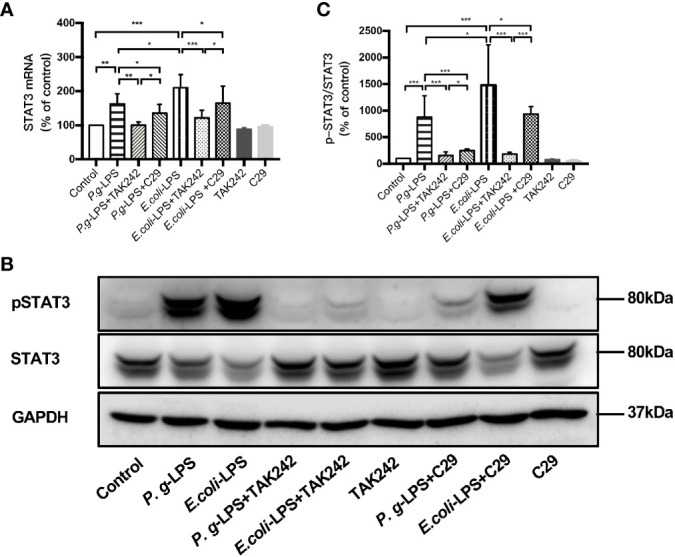
Different effects of *P. gingivalis*-LPS and *E. coli*-LPS on the gene, protein expression, and phosphorylation of STAT3 in BV−2 microglial cells. **(A)** BV-2 microglial cells were treated with TAK-242 (1μM), C29 (100μM), or serum-free medium for 60min, followed by treatment with *P. gingivalis*-LPS, *E. coli*-LPS (1μg/mL), or serum-free medium for 6h, and RT-PCR was performed. **(B)** BV-2 microglial cells were treated with TAK-242 (1μM), C29 (100μM) or serum-free medium for 60min, following by treatment with *P. gingivalis*-LPS, *E. coli*-LPS (1μg/mL) or serum-free medium for 6h and western blotting with anti-phosphorylated (p)-STAT3, anti-STAT3, and anti-GAPDH antibodies. **(C)** Semiquantitative analysis of the protein levels. Data from three independent experiments are presented as mean ± SD; Student’s *t*-test, *p < 0.05 compared the *P. gingivalis*-LPS group with the *E. coli*-LPS group; one-way ANOVA, *p < 0.05, **p < 0.01 and ***p < 0.001 compared to the control group, the LPS groups or the LPS plus TAK-242 groups.

Signal transducers and activators of transcription (STAT) can regulate the biological behavior of immune cells through inflammatory mediators and are key molecules in the formation of inflammation. In the STATs family, STAT3 is a key signaling molecule that induces neuroinflammatory responses following treatment with LPS. As shown in **Figures 6** and **7**, We performed RT-PCR, IF staining, and WB analysis to investigate the phosphorylation and nuclear translocation of STAT3 in *P. gingivalis*-LPS-stimulated BV-2 cells and further compared this with *E. coli*-LPS.

P-STAT3/STAT3 protein expression showed an increase in 6h after *P. gingivalis*-LPS stimulation and peaked at 6h (**Figures 6A, C**). To further validate these findings, we observed the phosphorylation and nuclear translocation of STAT3 under a fluorescent microscope and found that significant nuclear translocation was observed 6h after stimulation with *P. gingivalis*-LPS in BV-2 microglial cells (**Figures 6D, E**). In contrast, the *E. coli*-LPS group showed an increase of p-STAT3/STAT3 protein expression at 3h, which also peaked at 6h. Similarly, nuclear translocation of p-STAT3 was observed at 3h after beginning stimulation with *E. coli*-LPS (**Figures 6B–E**).

Moreover, the elevated expression of STAT3 mRNA and p-STAT3/STAT3 protein induced by *P. gingivalis*-LPS was attenuated by the TLR4 inhibitor TAK-242 or TLR2 inhibitor C29 at 6h. Compared with C29-treated groups, TAK-242-treated groups were more effective in reducing STAT3 mRNA and p-STAT3/STAT3 protein expression (**Figures 7A–C**). Meanwhile, significant differences in STAT3 mRNA and p-STAT3/STAT3 protein expression were observed between the *P. gingivalis*-LPS group and *E. coli*-LPS group at 6h (**Figures 7A–C**).

These results provide evidence for the involvement of *P. gingivalis*-LPS Standard in the downstream signal processing of TLR4 instead of TLR2 and the ability of *P. gingivalis*-LPS in the activation of the NF-κB/STAT3 signaling. However, *P. gingivalis*-LPS is a weaker NF-κB/STAT3 signaling activator than *E. coli*-LPS.

### Binding Mode Prediction of *P. gingivalis*-Lipid A and *E. coli*-Lipid A to TLR4-MD-2

In computer-assisted drug design methods, structure-based molecular docking is a well-known method used to predict the binding positions of ligands into the protein’s active site. In this study, Autodock Vina was used to dock and predict the binding mode and binding affinity of lipid As to TLR4-MD-2, and we further compared the different binding ability of *P. gingivalis* and *E. coli*-lipid A to TLR4-MD-2. The docking scores are shown in **Table 2**, in which a more negative score indicates a stronger binding affinity of a ligand to the TLR4-MD-2 receptor with more favorable intermolecular interactions. The docking score of *E. coli*-lipid A to TLR4-MD-2 was -38.3 kcal/mol with the root mean square deviation (RMSD) of 2.20 Å compared to the co-crystallized conformation of *E. coli*-lipid A, suggesting that the docking strategy applied was reasonable and reliable to sample the binding conformation of the ligand (Thomsen and Christensen, 2006).

**Table 2:** Comparison of binding free energies between lipid As of *E. coli* and *P. gingivalis* with TLR4-MD-2.

Ligand	Binding free energy (kcal/mol)	Fatty acids	RMSD (Å)
*E. coli*-lipid A	-38.3	6	2.2
5*P. g*-lipid A	-27.7	5	/
4a*P. g*-lipid A	-15.6	4	/
4b*P. g*-lipid A	-13.4	4	/
3*P. g*-lipid A	-12.5	3	/

The binding energies of 5*P. g*-Lipid A, 4a*P. g*-Lipid A, 4b*P. g*-Lipid A, and 3*P. g*-Lipid A to TLR4-MD-2 were -27.7, -15.6, -13.4, and -12.5 kcal/mol, respectively, which are weaker than the hexa-acylated *E. coli*-lipid A. Meanwhile, the binding affinities of the four *P. gingivalis*-lipids As to TLR4-MD-2 were almost linear to their number of acylated lipid chains.

### Binding Mode Analysis of *P. gingivalis*-Lipid A and *E. coli*-Lipid A to TLR4-MD-2

**Figure 8:**
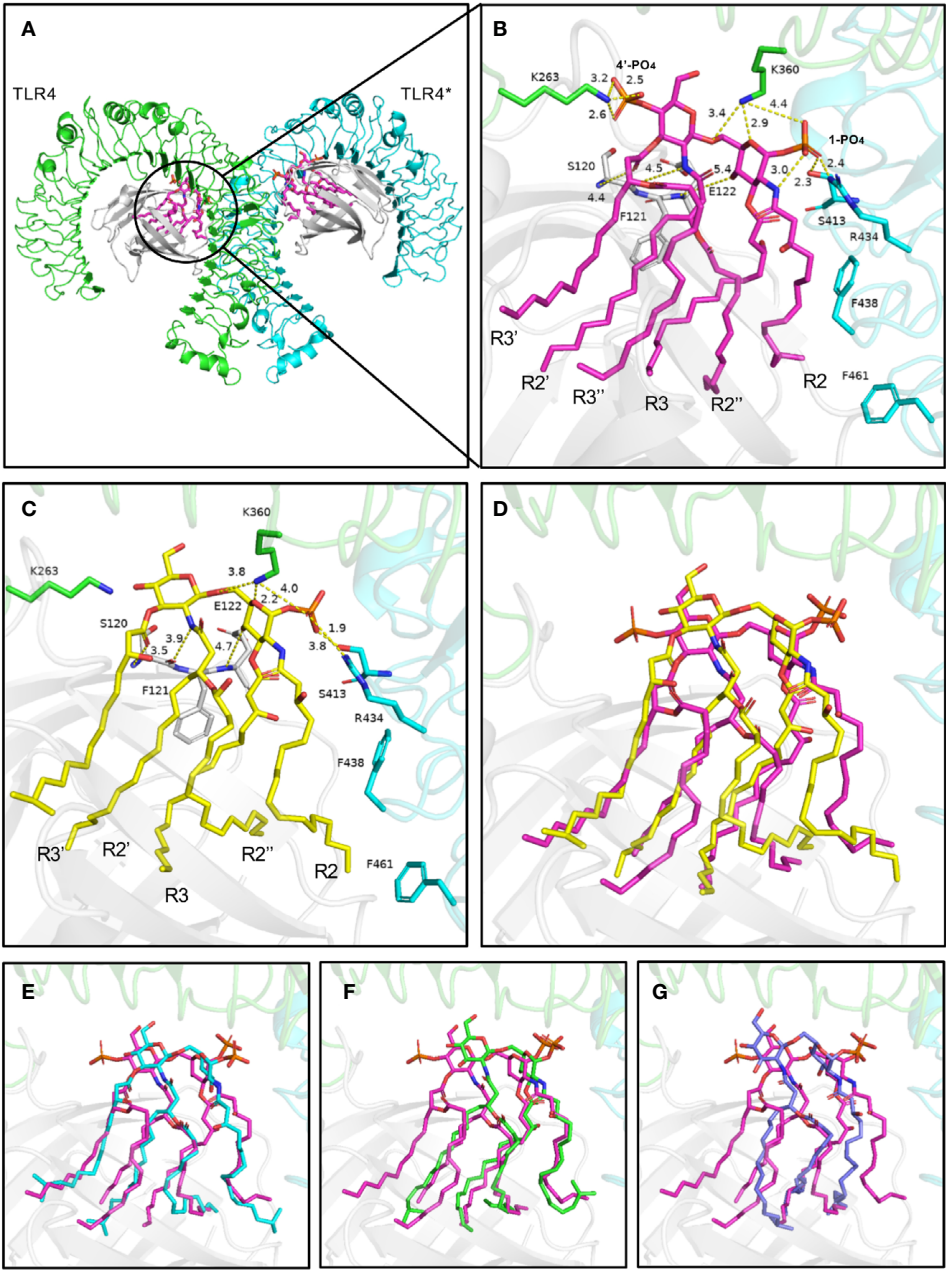
Binding modes of lipid A from *E. coli* **(A, B)** and *P. g* **(C**–**G)** with TLR4-MD-2. **(A)** Top view of the symmetrical dimer of the TLR4-MD-2-LPS complex (PDB 3VQ2). **(B)** Hydrogen bond interactions of the 1-phosphate and 4’-phosphate residues of lipid A. Interaction distance in Ångstrom are indicated. **(C, D)** The predicted binding modes of 5*P. g*-lipid A, **(E)** 4a*P. g*-lipid A, **(F)** 4b*P. g*-lipid A, and **(G)** 3*P. g*-lipid A with the receptor. *E. coli*-lipid A is shown as magenta sticks, while the four types of *P. g*-lipid A are displayed as yellow, cyan, green, and lightblue sticks, respectively. Key residues are shown as sticks. Hydrogen bonds are depicted as yellow dashed lines. TLR4, TLR4*, MD-2 are colored green, cyan, and gray, respectively.

The TLR4-MD-2 complex was dimerized, driven by LPS (**Figures 8A, B**). In the crystal structure, the lipid chains of *E. coli*-lipid A binds at the hydrophobic pocket in MD-2 (Ohto et al., 2012) with five of the six acyl chains (R3, R2’, R3’, R2’’, and R3’’) completely buried inside the pocket, and the remaining R2 chain partially exposed on the surface of MD-2 (**Figures 8A, B**). The hydrophobic R2 chain forms hydrophobic interactions with residues F438* and F461* (* represent another TLR4 molecule). The favorable intermolecular interactions formed by the hydrophobic R2 chain at the interface are important for dimerization of the TLR4-MD-2 complex (Park et al., 2009; Ohto et al., 2012; Artner et al., 2013; Maeshima and Fernandez, 2013). The 1-phosphate, at the dimerization interface, also dramatically affects dimerization by forming ionic and hydrogen bond interactions with TLR4 (Park et al., 2009; Ohto et al., 2012). The positively charged cluster of R434*, S413*, and K360 could form hydrogen bonds with 1-PO_4_. Similarly, K263 of TLR4 could form hydrogen bonds with 4’-PO_4_. Meanwhile, the 4-hydroxyl group, 2’-imino group, and 3’-carbonyl group of glucosamine contribute to the intermolecular interaction by forming hydrogen bonds with backbones of E122, F121 and S120 of MD-2, respectively (**Figures 8A, B**).

The binding modes of tri-, tetra-, and penta-acylated *P. gingivalis*-lipid A with TLR4-MD-2 were simulated by molecular docking and were analyzed based on the above discussed key intermolecular interactions of *E. coli*-lipid A and TLR4-MD-2. Generally, the four *P. gingivalis*-lipid As all bind at the hydrophobic pocket in MD-2 like that of *E. coli*-lipid A. The R2 chain of 5*P. g*-Lipid A maintains the same conformation as *E. coli*-lipid A (**Figures 8C, D**). The decreased binding affinity of 5*P. g*-Lipid A may be attributed to the lack of an R3’’ chain and a 4’-phosphate group, which could form hydrophobic interactions and hydrogen bonds with MD-2 and TLR4, respectively. Similarly, R2 chains of the two tetra-acylated *P. gingivalis*-lipid As also matched the one in *E. coli*-lipid A well (**Figures 8E, F**). However, due to the lack of R3, and R3’’ or R3’and R3’’, the binding energies of the two tetra-acylated *P. gingivalis*-lipid As were inferior to that of *E.coli*-lipid A or 5*P.g*-Lipid A. Compared to the above three *P. gingivalis*-Lipid As, the tri-acylated *P. gingivalis*-lipid A adopted a slightly shifted binding conformation (**Figure 8G**), especially for the R2 chain. The less acylated lipid chains of 3*P. g*-Lipid A could formless hydrophobic interactions with the receptor, thus resulting in the weakest binding energies among the four *P. gingivalis*-lipid As.

Although the acyl chains of Lipid As from *P. gingivalis* are 2~4 C atoms longer than those of *E. coli*, they all could fully extend in the pocket, suggesting that the hydrophobic pocket in MD-2 is large enough to accommodate diverse Lipid As. Besides, compared to the *E. coli*-lipid A, the diglucosamine backbone of *P. gingivalis*-lipid As containing four and five fatty acids does not move upward obviously with the 1-phosphate group that is oriented almost in the same position to form electrostatic interactions with the positively-charged residues at the dimerization interface. These hydrophobic interactions in the pocket and polar electrostatic interactions at the mouth of the MD-2 pocket between the *P. gingivalis*-lipid As and the receptor will collectively contribute to the bioactivities of the *P. gingivalis*-lipid As towards TLR4. These results indicate that the different acyl chains and phosphate groups have differential signaling effects on the bioactivity of TLR4, which may be the reason for the experimentally observed weaker TLR4 activating properties of *P. gingivalis*-LPS.

## Discussion

In this study, we found that *P. gingivalis*-LPS could upregulate the gene expression and secretion of pro-inflammatory factors IL-1β, IL-6, TNF-α, IL-17, and IL-23 in BV2 microglial cells. Meanwhile, increased levels of TLR2/4 were observed, following the activation of NF-kB/STAT3 signaling. In contrast, the changes mentioned above were more significant in the *E. coli*-LPS group, while the effects of both types of LPS could be reversed by the TLR2 inhibitor C29 and TLR4 inhibitor TAK-242. Furthermore, the molecular docking showed that *E. coli*-lipid A had a stronger binding affinity than *P. gingivalis*-lipid A with the TLR4-MD-2 receptor. Our findings indicated that *P. gingivalis*-LPS is a weaker TLR2/4 agonist and NF-κB/STAT3 signaling activator, and the composition of the different fatty acid chains and phosphate groups between *P. gingivalis*-lipid A and *E. coli*-lipid A may be the cause of these differences.

*E. coli*-LPS has been proven to induce upregulation of inflammatory cytokines in BV-2 cells by activating multiple signaling pathways such as Akt/STAT3 (Nam et al., 2018). NF−κB and STAT3 are transcription factors that play a crucial role in neuroinflammatory responses (Park et al., 2020). NF−κB and STAT3 phosphorylate, translocate to the nucleus, and bind to the enhancers in the inflammatory cytokines promoter to induce gene transcription (Park et al., 2020). According to our findings, *P. gingivalis*-LPS could activate the NF-κB/STAT3 signaling pathway in BV-2 microglia cells—increasing the phosphorylation status of NF-κB and STAT3 proteins and inducing their nuclear translocation—leading to up-regulation of IL-1β, IL-6, TNF-α, IL-23, and IL-17A inflammatory cytokine gene expression. Compared with *E. coli*-LPS, *P. gingivalis*-LPS induces a weaker modulation of inflammatory cytokine gene expressions by differentially activating the NF-κB/STAT3 signaling pathway.

TLRs are type I transmembrane proteins that recognize pathogen-derived macromolecules and play a key role in the innate immune system. These pathogen-derived macromolecules are broadly shared by pathogens but distinguishable from the host molecules, and they are collectively referred to as pathogen-associated molecular patterns (PAMPs) (Takeda and Akira, 2004). TLRs are mainly expressed in immune cells and have also been identified in diverse CNS cell types, such as microglia (Hanke and Kielian, 2011). TLR4 is the most representative member of the TLRs family and predominantly responds to LPS through its co-receptor, myeloid differentiation protein-2 (MD-2), which is essential for LPS-induced stimulation of TLR4 (Shimazu et al., 1999). Some early studies indicated that LPS mediated immune-inflammatory responses through both TLR2 and TLR4 (Muta and Takeshige, 2001; Su et al., 2015). However, recent reports showed that LPS-dependent signals are transmitted through TLR4 and not TLR2 (Nativel et al., 2017), while it is the lipoproteins/lipopeptides (LPs) from the outer membrane of bacterial that activate TLR2 (TLR2 associated with TLR1 or TLR6) but not LPS (Satoh and Akira, 2016). In our study, we chose standard LPS preparations containing other bacterial components, such as lipoproteins, and therefore can stimulate both TLR2 and TLR4. The results showed that *P. gingivalis*-LPS and *E. coli*-LPS preparations could simultaneously upregulate the mRNA and protein expression of TLR2/4 and activate TLR2/4-mediated inflammatory signaling pathways. Moreover, inhibitors of TLR2 (C29) and TLR4 (TAK-242) could block the elevated expression of TLR2 or TLR4-mediated transcription factors and inflammatory cytokine, respectively. In the **Supplementary Figures S1–S3**, we further added TLR4-specific ligand (LPS-Ultrapure), TLR2-specific ligands (Pam3CSK4), and heat-inactivated strains to observe the effect of immune-inflammatory responses in BV-2 microglial cells and also to demonstrate the effect of TLR2/4 small molecule inhibitors. The results indicated that both TLR2 and TLR4 are involved in the immuno-inflammatory responses in BV-2 microglial cells induced by heat-inactivated *P. gingivalis*, while the immune-inflammatory responses induced by LPS-Ultrapure or Pam3CSK4 were only involved in TLR4 or TLR2, respectively.

It is well known that CD14 is an important co-receptor for TLR4 to bind to LPS and induce signaling cascades (Wright et al., 1990). CD14/LPS interacts with TLR4 to transfer LPS to TLR4/MD2. Two LPS-bound TLR4/MD2 complexes form an M-shaped dimer followed by activation of the signaling pathway for the innate immune response (Kim and Kim, 2017; Ryu et al., 2017). It has been reported that CD14 mediates cellular responses depending on the concentration of LPS, and at low concentrations of LPS (1-10ng/ml), CD14 was indispensable for its activation of cells, while at higher concentrations (100-1000ng/ml), CD14 provides a partial contribution (Borzęcka et al., 2013). Yang et al. (2016) revealed that 5μg/ml LPS can upregulate CD14 gene expression in alveolar macrophages (AM). Hu et al. (2020) found that TLR4 and CD14 mRNA and protein expressions were upregulated in the peripheral blood mononuclear cells (PBMC) and cerebral cortex of rats with periodontitis established by gingival sulcus injection of *P. gingivalis*-LPS, and could be reversed by TAK-242. Our experiments also revealed that CD14 mRNA and protein expression were upregulated in 1μg/mL LPS-stimulated BV-2 cells and could be reversed by TAK-242, indicating a partial role for CD14 in the recognition of high concentrations of LPS by TLR4, and the expression of CD14 is also affected by LPS-TLR4.

The structure of LPS includes lipid A, a core oligosaccharide, and an O-antigen. As the main active component of LPS, lipid A can bind to MD2 to induce the dimerization of TLR4, and thus activating the downstream signaling pathways which then lead to the secretion of pro-inflammatory cytokines (Xiao et al., 2017). Differences are observed in lipid A structures from different Gram-negative bacteria, and it is well established that subtle changes in the chemical structure can result in dramatically different immune activities: the numbers, types, and positions of the fatty acid chains govern the immunological activity of LPS, and the total number of lipid chains is the most important factor (Rietschel et al., 1993; Rietschel et al., 1994; Coats et al., 2009).

Ogawa et al. (2007) were successful in extracting LPS of *P. gingivalis*, which was recognized to contain tri-, tetra-, and penta-acylated components. These structures differ from hexa-acylated *E. coli*-lipid A in the number of phosphates and the numbers, types, positions of the fatty acid chains. Specifically, the number of lipid chains of *P. gingivalis*-lipid A is less than those of *E. coli*-lipid A, but the lipid chain of *P. gingivalis* is 2~4 C atoms longer than that of *E. coli* (**Figure 1**). As shown by (Herath et al. 2011; 2013), tetra- and penta-acylated lipid A structures of *P. gingivalis*-LPS differentially activated the TLR4-mediated NF-κB signaling pathway, and the expression of inflammatory cytokines was significantly upregulated by penta-acylated lipid A while they were downregulated by tetra-acylated lipid A in human gingival fibroblasts (HGFs). According to our docking, at the dimerization interface, the hydrophobic R2 acyl chains could affect lipid A’s recognition by TLR4. Compared to the *E. coli*-lipid A, the glucosamine located in *P. gingivalis*-lipid A did not move up obviously. The LPS having five or fewer lipid chains could not adequately fill the large empty hydrophobic pocket in MD-2 well, thus leading to reduced binding affinities. Meanwhile, the two phosphate groups in the lipid A region also play an important role in the endotoxic activity of LPS. The absence of one of these phosphate groups could cause an about 100-fold decrease in activity (Rietschel et al., 1993; Rietschel et al., 1994; Coats et al., 2009). According to the reported structures, lipid A of *P. gingivalis* is monophosphoryl at 1-position while lipid A of *E. coli* is diphosphoryl at both 1- and 4’- positions. The lack of a 4’-PO_4_ of *P. g*-Lipid A means that it would be unable to form additional hydrogen bond interactions with a positively charged lysine from TLR4, similar to that of *E. coli*-lipid A, which may be another reason for the lower binding affinity of *P. gingivalis*-Lipid A than *E. coli*-lipid A.

Lipoproteins are important constituents of the bacterial cell envelope and are characterized by the presence of conserved N-terminal lipid-modified cysteine residues, allowing hydrophilic proteins to anchor onto bacterial cell membranes. Moreover, they can induce innate immune reactions by functioning as ligands of the mammalian TLR2 (Braun and Hantke, 2019). Herein, we found different activation potencies of the *P. gingivalis*-LPS and *E. coli*-LPS preparations against TLR2, and the different structure of lipoproteins in the LPS preparations between *P. gingivalis* and *E. coli* may be the reason. *P. gingivalis*-lipoprotein (PG1828) and *E. coli*-lipoprotein (LPP) are both tri-acylated N-terminal structures, but the lengths of the acylated fatty chains on the cysteine residue are different (Nakayama et al., 2012). Because of the diverse combinations of the fatty acid chains, and the fact that phenol-water extraction method is only used to obtain LPS instead of lipoproteins, the structure of the lipoproteins in LPS preparations cannot be determined, and we cannot use the molecular docking method to analyze the reason for the differential activation of TLR2. To enable thorough studies on lipoproteins, we may try to enrich bacterial lipoproteins and prepare N-terminal trypsin lipopeptides, and perform structure determination by matrix-assisted laser desorption ionization time-of-flight mass spectrometry (MALDI-TOF MS) in the future (Armbruster and Meredith, 2018).

## Conclusion

This study suggests that *P. gingivalis*-LPS preparation could activate TLR2/4-mediated NF-κB/STAT3 signaling pathways, which ultimately result in the immune-inflammatory response of BV-2 microglia. In contrast to *E. coli*-LPS, *P. gingivalis*-LPS preparation is a weaker TLR2/4 agonist and NF-κB/STAT3 signaling activator. Furthermore, the different fatty acid chains and phosphate groups between *P. gingivalis*-lipid A and *E. coli*-lipid A may be the reason for the weaker activating properties of *P. gingivalis*-LPS preparation.

## Data Availability Statement

The raw data supporting the conclusion of this manuscript will be made available by the authors, without undue reservation.

## Author Contributions

All the authors contributed to the writing of this manuscript. CQ, ZY, SL, WZ, and ZS conceptualized the study. CQ, ZY, ZH, YL, and HC curated the data, conducted a formal analysis, and reviewed and edited the article. WZ and ZS acquired funding. CQ and ZY worked on the methodology. CQ, ZY, ZH, YL, and HC administered the project. SL, WZ, and ZS supervised the study. All authors contributed to the article and approved the submitted version.

## Funding

This work was supported by the National Natural Science Foundation of China (NSFC) (No. 81971299 and 82071112), the Science and Technology Commission of Shanghai Municipality (STCSM) (project number: 20ZR1431800), and the Cross-disciplinary Research Fund of Shanghai Ninth People’s Hospital, Shanghai Jiao Tong University School of Medicine (JYJC202005).

## Conflict of Interest

The authors declare that the research was conducted in the absence of any commercial or financial relationships that could be construed as a potential conflict of interest.
