# Supplementary material for: Lipopolysaccharide Preparation Derived From Porphyromonas gingivalis Induces a Weaker Immuno-Inflammatory Response in BV-2 Microglial Cells Than Escherichia coli by Differentially Activating TLR2/4-Mediated NF-κB/STAT3 Signaling Pathways
Source: Front Cell Infect Microbiol. 2021 Mar 18;11:606986. doi: 10.3389/fcimb.2021.606986 (PMC8012810; doi:10.3389/fcimb.2021.606986)
Supplement: Supplementary file 1 [file DataSheet_1.pdf]

Supplementary Material

1 Materials and methods

1.1 TLR2- and TLR4- specific ligands

TLR4 Agonist (LPS-Ultrapure) from *P. gingivalis* or *E. coli* 055: B5 was purchased from InvivoGen (San Diego, CA, USA) and TLR2/TLR1 Agonist (Pam3CSK4) was purchased from Abmole (Houston, TX, USA).

1.2 Bacterial culture condition and inactivation

P. gingivalis (ATCC 33277) and *E. coli* (ATCC 25922) were provided by the Shanghai Research Institute of Stomatology and Shanghai Key Laboratory of Stomatology, Shanghai Ninth People's Hospital (Shanghai, China). *P. gingivalis* were maintained in Brain Heart Infusion (BHI, BD Bioscience, Sparks, MD, USA) and Brain Heart Infusion Agar (BHI Agar, BD Bioscience), while *E. coli* was maintained in Lysogeny Broth (LB, BD Bioscience) and Lysogeny Broth Agar (LB Agar, BD Bioscience). In addition, 10μg/mL vitamin K and 5μg/mL hemin were added to the culture medium of *P. gingivalis*. For heat-killing the bacteria, the prepared bacteria were placed in 76°C heating block for 1h. Heat-killed bacteria were incubated with BV-2 microglia cells at a multiplicity of infection (MOI) of 100.

1.3 Reverse-transcription and real-time PCR

After different simulation, total RNA was extracted from BV-2 microglia cells with E.Z.N.A.® Total RNA Kit I (Omega Bio-tek, Georgia, USA) according to the manufacturer's instructions. A total of 1000ng of extracted RNA was reverse-transcribed to cDNA using a PrimeScript™ RT reagent Kit (Takara, Otsu, Shiga, Japan). The primer sequences specific to IL-1β, IL-6, TNF-α, IL-17A, IL-23, and β-actin for BV-2 microglial cells are shown in **Table 1**. Real-time PCR was performed in a LightCycler480 system (Roche, Basel, Switzerland) using TB Green® Premix Ex Taq™ (Takara, Otsu, Shiga, Japan). The DNA amplification was performed as follows: the first cycle was maintained at 95°C for 30s, followed by 40 cycles consisting of denaturation (95°C for 10s), annealing and extension (60°C for 30s). The values obtained for the target gene expression were normalized to β-actin and quantified relative to the expression in control samples using the $2^{-\Delta\Delta Ct}$ method.

1.4 Enzyme-linked immunosorbent assay (ELISA)

To explore the effects of Ultrapure-LPS, Pam3CSK4 and heat-killed bacteria on IL-1β, IL-6, and TNF-α levels in BV-2 microglial cells, an enzyme-linked immunosorbent assay (ELISA) was performed. Briefly, BV-2 microglial cells were pre-treated with TAK-242 (1μM) or C29 (100μM) for 60min, then treated with Standard-LPS (1μg/mL), Ultrapure-LPS (1μg/mL), Pam3CSK4 (1μg/mL) or heat-killed bacteria (MOI of 100) for 6h. ELISA was used to measure levels of pro-IL-1β, mature-IL-1β, IL-6, and TNF-α using the conditioned medium (mature-IL-1β, IL-6, and TNF-α) or cell homogenate (pro-IL-1β). Mouse IL-1β, IL-6, and TNF-α ELISA kits (NeoBioscience, Shenzhen, China) were used according to the manufacturer's recommendations. The absorbance of the samples was measured at 450 nm using a microplate reader (Epoch2, Bio-Tek, Winooski, VT, USA).

1.5 Western blotting

To explore Ultrapure-LPS, Pam3CSK4 and heat-killed bacteria effects on NF-κB signaling in BV-2 microglial cells, BV-2 microglial cells were treated with TAK-242 (1μM) or C29 (100μM) for 60min, followed by Ultrapure-LPS (1μg/mL), Pam3CSK4 (1μg/mL) or heat-killed bacteria (MOI of 100) stimulation. After the final incubation, the cells were lysed with RIPA buffer containing 2% protease and phosphatase inhibitor cocktail (50mM, Beyotime Biotechnology) and 1% PMSF (100mM, Beyotime Biotechnology). Following the addition of sodium dodecyl sulfate (SDS) loading buffer, the samples were boiled for 5min, and proteins were subsequently detected by western blotting analysis. The proteins were transferred to a polyvinylidene difluoride (PVDF) membrane after separation. A wide range of protein markers was run in parallel to detect the molecular weight of proteins. A 5% skimmed milk solution was used for membrane blockage to reduce nonspecific binding. Proteins were probed with specific antibodies and images were quantified using ImageJ 1.52a software (National Instituted of Health, Bethesda, MD, USA).

2 Result

2.1 Effects of Ultrapure-LPS on the expression of Inflammatory cytokine and phosphorylation of NF-κB p65 in BV-2 microglial cells.

After 1μg/mL Ultrapure-LPS simulation for 6h, the *P. gingivalis*-LPS Ultrapure group showed an approximately 1.30-, 1.37-, or 1.25-fold increase in IL-1β, IL-6, or TNF-α gene upregulation in comparison with the control group, respectively (**Figure 1S A-C**). Meanwhile, the expression of pro-IL-1β, IL-6, and TNF-α protein in the *P. gingivalis*-LPS Ultrapure group was significantly higher than that in the control group (**Figure 1S D-F**). In contrast, the *E. coli*-LPS Ultrapure group showed an

approximately 85-, 57-, or 16-fold increase in IL-1β, IL-6, or TNF-α gene upregulation compared with the control group, and the expression of pro-IL-1β, IL-6, and TNF-α protein in the *E. coli*-LPS Ultrapure group was significantly higher than that of the control group. Significant differences were observed between the *P. gingivalis*-LPS Ultrapure group and *E. coli*-LPS Ultrapure group as mentioned above (**Figure 1S A-F**). Furthermore, the Ultrapure-LPS plus TAK-242 groups showed downregulation of IL-1β, IL-6, TNF-α mRNA and protein expression in comparison to the Ultrapure-LPS group (**Figure 1S A-F**).

Moreover, the elevated expression of phosphor(p)-p65/p65 protein induced by LPS-Ultrapure was attenuated by the TLR4 inhibitor TAK-242 rather than the TLR2 inhibitor C29 at 6h (**Figure 1S G**). Meanwhile, significant differences of p-p65/p65 protein expression were observed between the *P. gingivalis*-LPS Ultrapure group and *E. coli*-LPS Ultrapure group at 6h (**Figure 1S G**).

2.2 Effects of Pam3CSK4 on the expression of Inflammatory cytokine and phosphorylation of NF-κB p65 in BV-2 microglial cells.

After 1μg/mL Pam3CSK4 simulation for 6h, the Pam3CSK4 group showed an approximately 60-, 57-, or 15-fold increase in IL-1β, IL-6, or TNF-α gene upregulation in comparison with the control group, respectively (**Figure 2S A-C**). Meanwhile, the expression of pro-IL-1β, IL-6, and TNF-α protein in the Pam3CSK4 group was significantly higher than that in the control group (**Figure 2S D-F**). Furthermore, the Pam3CSK4 plus C29 groups showed downregulation of IL-1β, IL-6, TNF-α mRNA and protein expression in comparison to the Pam3CSK4 group (**Figure 2S A-F**).

Moreover, the elevated expression of p-p65/p65 protein induced by Pam3CSK4 was attenuated by the TLR2 inhibitor C29 rather than the TLR4 inhibitor TAK-242 at 6h (**Figure 2S G**).

2.3 Effects of heat-killed bacteria on the expression of Inflammatory cytokine and phosphorylation of NF-κB p65 in BV-2 microglial cells.

After heat-killed bacteria (MOI of 100) simulation for 6h, the heat-killed *P. gingivalis* group showed an approximately 13-, 34-, or 3-fold increase in IL-1β, IL-6, or TNF-α gene upregulation in comparison with the control group, respectively (**Figure 3S A-C**). Meanwhile, the expression of pro-IL-1β, IL-6, and TNF-α protein in the heat-killed *P. gingivalis* group was significantly higher than that in the control group (**Figure 3S D-F**). In contrast, the heat-killed *E. coli* group showed an approximately 238-, 247-, or 10-fold increase in IL-1β, IL-6, or TNF-α gene upregulation compared with the control group, and the expression of pro-IL-1β, IL-6, and TNF-α protein in the heat-killed *E. coli* group was significantly

higher than that of the control group. Significant differences were observed between the heat-killed *P. gingivalis* group and heat-killed *E. coli* group as mentioned above (**Figure 3S A-F**). Furthermore, the heat-killed bacteria plus TAK-242 groups and heat-killed bacteria plus C29 groups showed downregulation of IL-1β, IL-6, TNF-α mRNA and protein expression in comparison to the heat-killed bacteria group. (**Figure 3S A-F**).

Moreover, the elevated expression of p-p65/p65 protein induced by heat-killed bacteria was attenuated by the TLR4 inhibitor TAK-242 or TLR2 inhibitor C29 at 6h (**Figure 3S G**). Meanwhile, significant differences of p-p65/p65 protein expression were observed between the heat-killed *P. gingivalis* group and heat-killed *E. coli* group at 6h (**Figure 3S G**).

2.4 Gene expression of inflammatory cytokines in Standard LPS-stimulated BV-2 microglial cells

As shown in **Figure 4S**, RT-PCR assays were performed for IL-1β, IL-6, TNF-α, IL-17A, and IL-23 genes. The results indicate that IL-1β, IL-6 and TNF-α mRNA expression peaked at 6h after Standard -LPS stimulation, while IL-17A or IL-23 mRNA expression peaked at 24h or 12h, respectively.

2.5 Mature-IL-1β release in Standard LPS-stimulated BV-2 microglial cells

As shown in **Figure 5S**, ELISA was used to measure levels of mature-IL-1β using the cell medium. The result indicates that mature IL-1β was not visible in cell medium.

3 Supplementary Figures

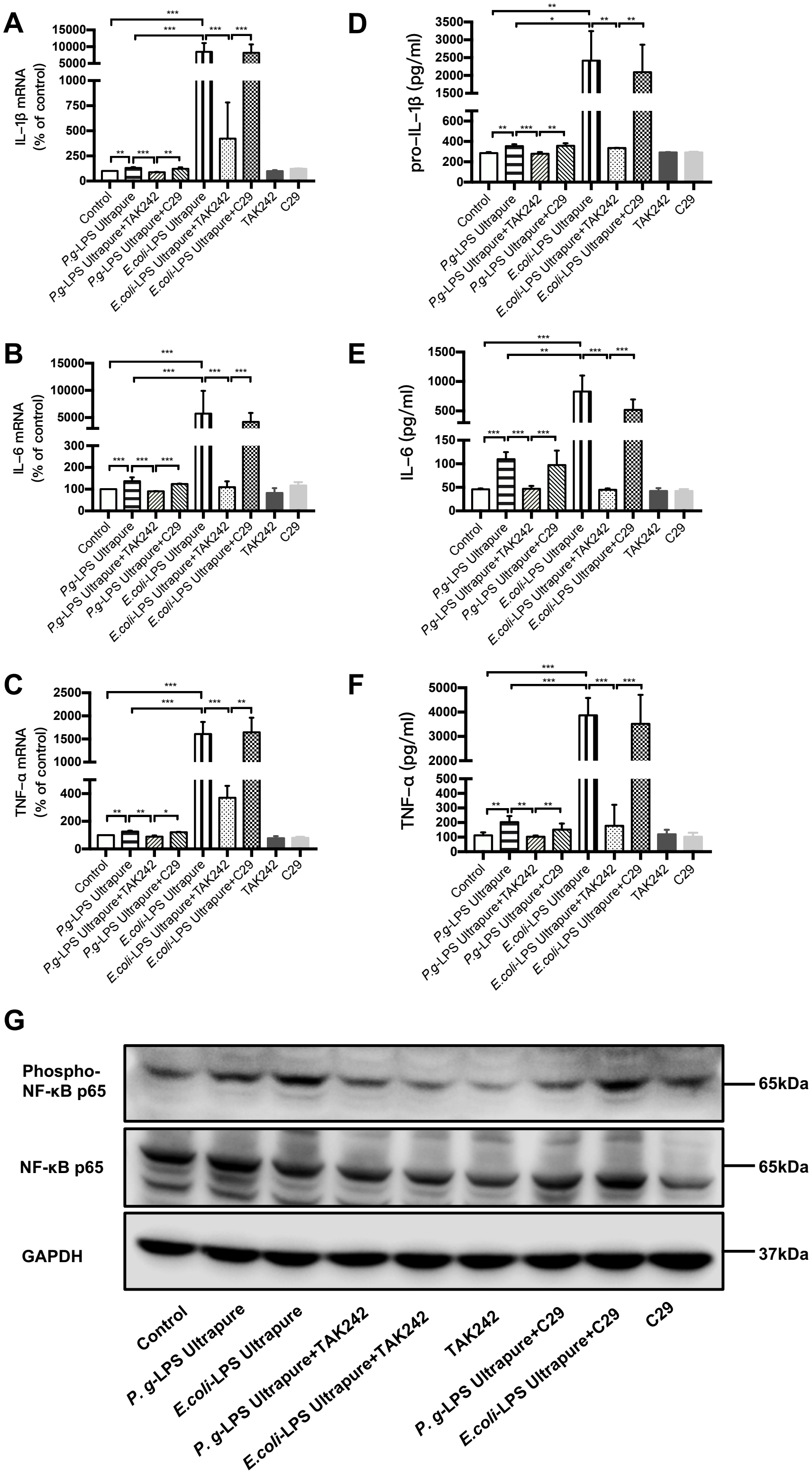

Figure S1. Inflammatory cytokine expression and phosphorylation of NF-κB p65 in Ultrapure-LPS stimulated BV-2 microglial cells. (**A-C**) BV-2 microglial cells were treated with TAK-242 (1μM), C29 (100μM), or serum-free medium for 60min, followed by treatment with Ultrapure-LPS (1μg/mL) or serum-free medium for 6h, and RT-PCR was performed. (**D-F**) BV-2 microglial cells were treated with TAK-242 (1μM), C29 (100μM) or serum-free medium for 60min, following by treatment with Ultrapure-LPS (1μg/mL) or serum-free medium for 6h and pro-IL-1β, IL-6, and TNF-α levels were measured using ELISA kits. (**G**) BV-2 microglial cells were treated with TAK-242 (1μM), C29 (100μM), or serum-free medium for 60min, followed by treatment with Ultrapure-LPS (1μg/mL) or serum-free medium for 6h and western blotting with anti-phosphorylated(p)-NF-κB p65, anti-NF-κB p65, and anti-GAPDH antibodies. Data from three independent experiments are presented as mean ± SD; Student's *t*-test, *p<0.05, **p<0.01, and ***p<0.001 compared the *P. gingivalis*-LPS Ultrapure group with the *E. coli*-LPS Ultrapure group; one-way ANOVA, *p<0.05, **p<0.01, and ***p<0.001 compared to the control group, the Ultrapure-LPS groups or the Ultrapure-LPS plus TAK-242 groups.

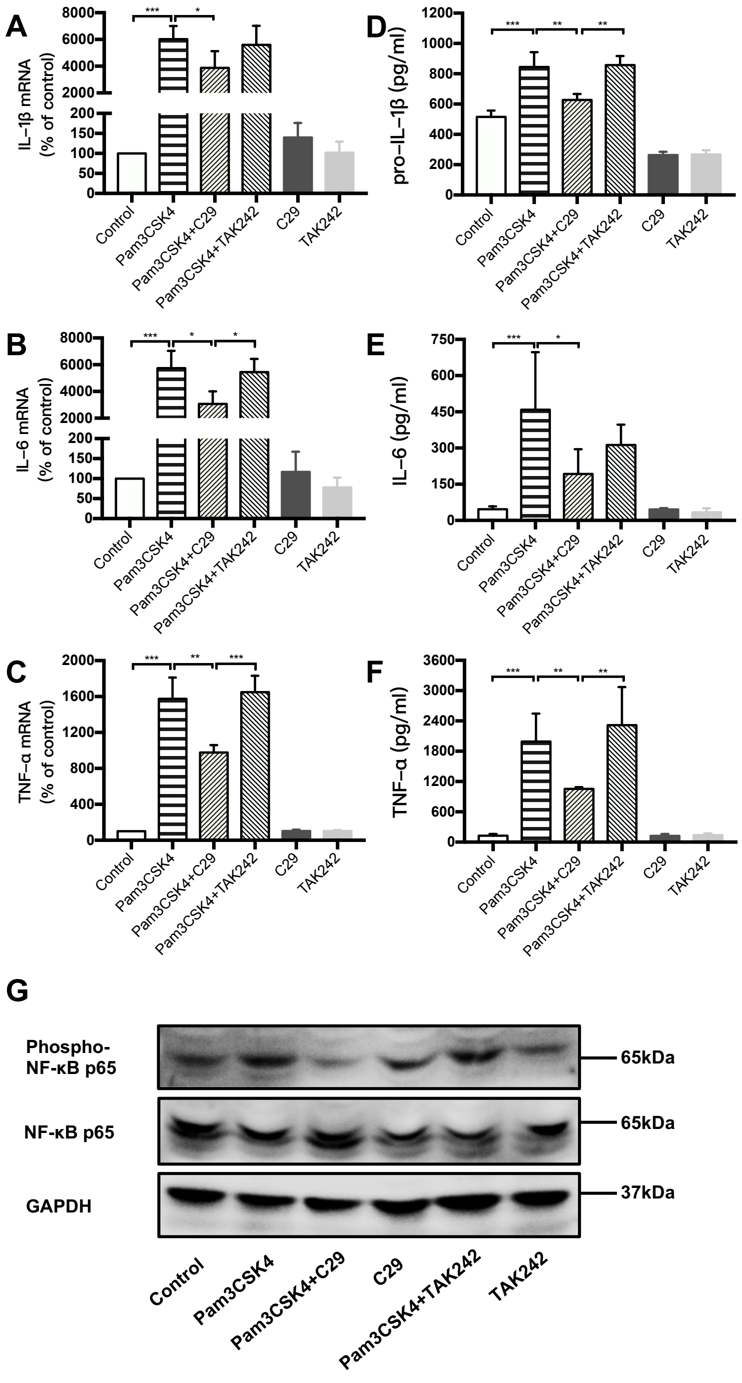
A
IL−1β mRNA (% of control)
B
IL−6 mRNA (% of control)
C
TNF−α mRNA (% of control)
D
pro−IL−1β (pg/ml)
E
IL−6 (pg/ml)
F
TNF−α (pg/ml)
Control
Pam3CSK4
Pam3CSK4+C29
Pam3CSK4+TAK242
C29
TAK242
G
Phospho-NF-κB p65
NF-κB p65
GAPDH
65kDa
65kDa
37kDa

Figure S2. Inflammatory cytokine expression and phosphorylation of NF-κB p65 in Pam3CSK4 stimulated BV-2 microglial cells. (**A-C**) BV-2 microglial cells were treated with TAK-242 (1μM), C29 (100μM), or serum-free medium for 60min, followed by treatment with Pam3CSK4 (1μg/mL) or serum-free medium for 6h, and RT-PCR was performed. (**D-F**) BV-2 microglial cells were treated with TAK-242 (1μM), C29 (100μM) or serum-free medium for 60min, following by treatment with Pam3CSK4 (1μg/mL) or serum-free medium for 6h and pro-IL-1β, IL-6, and TNF-α levels were measured using ELISA kits. (**G**) BV-2 microglial cells were treated with TAK-242 (1μM), C29 (100μM), or serum-free medium for 60min, followed by treatment with Pam3CSK4 (1μg/mL) or serum-free medium for 6h and western blotting with anti-p-NF-κB p65, anti-NF-κB p65, and anti-GAPDH antibodies. Data from three independent experiments are presented as mean ± SD; one-way ANOVA, *$p<0.05$, **$p<0.01$, and ***$p<0.001$ compared to the control group, the Pam3CSK4 groups or the Pam3CSK4 plus C29 groups.

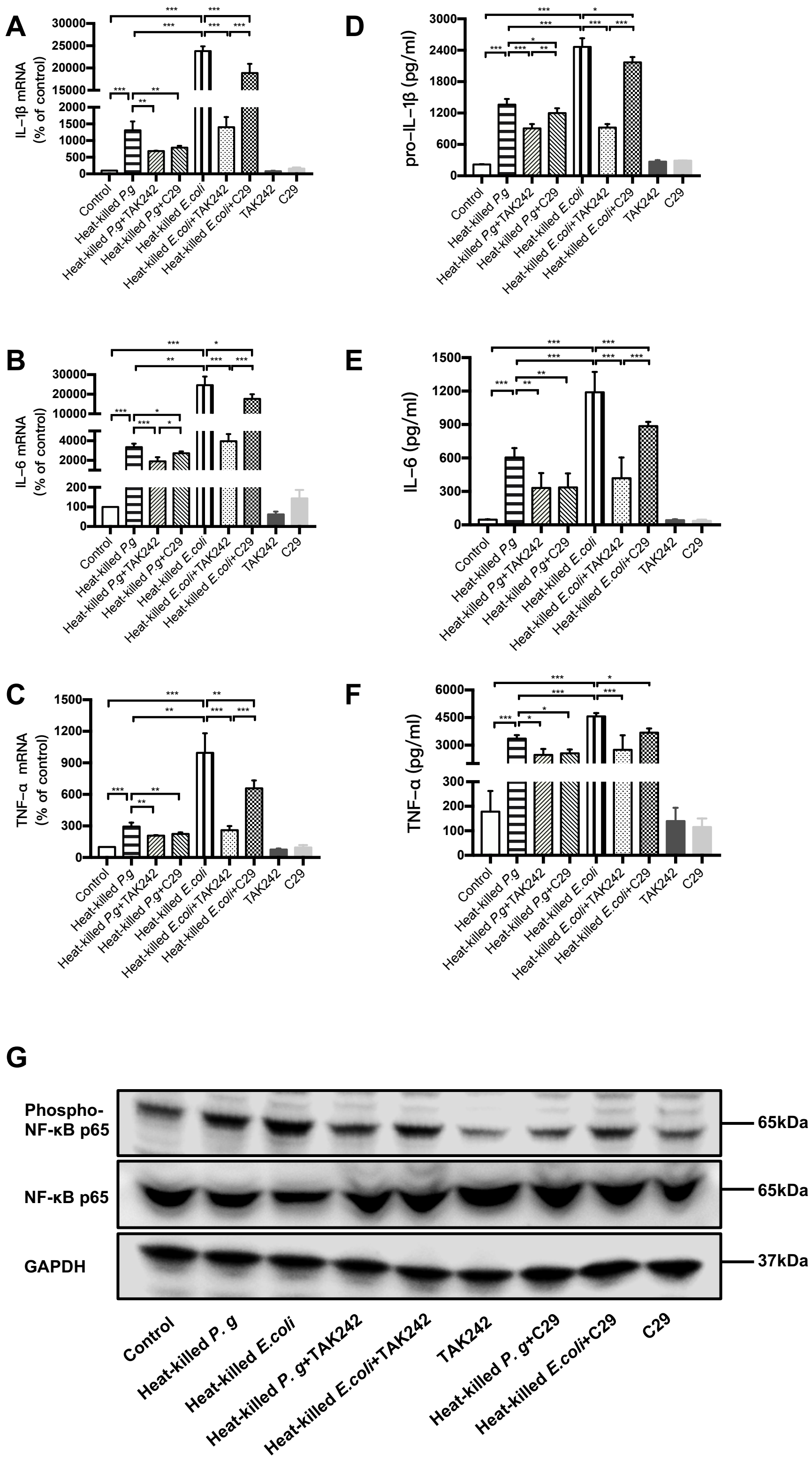

A
IL-1β mRNA (% of control)
B
IL-6 mRNA (% of control)
C
TNF-α mRNA (% of control)
D
pro-IL-1β (pg/ml)
E
IL-6 (pg/ml)
F
TNF-α (pg/ml)
Control
Heat-killed P.g
Heat-killed P.g+TAK242
Heat-killed P.g+C29
Heat-killed E.coli
Heat-killed E.coli+TAK242
Heat-killed E.coli+C29
TAK242
C29
G
Phospho-NF-κB p65
NF-κB p65
GAPDH
65kDa
65kDa
37kDa
Control
Heat-killed P. g
Heat-killed E.coli
Heat-killed P. g+TAK242
Heat-killed E.coli+TAK242
TAK242
Heat-killed P. g+C29
Heat-killed E.coli+C29
C29

Figure S3. Inflammatory cytokine expression and phosphorylation of NF-κB p65 in heat-killed bacteria stimulated BV-2 microglial cells. (**A-C**) BV-2 microglial cells were treated with TAK-242 (1μM), C29 (100μM), or serum-free medium for 60min, followed by treatment with heat-killed bacteria (MOI of 100) or serum-free medium for 6h, and RT-PCR was performed. (**D-F**) BV-2 microglial cells were treated with TAK-242 (1μM), C29 (100μM) or serum-free medium for 60min, following by treatment with heat-killed bacteria (MOI of 100) or serum-free medium for 6h and pro-IL-1β, IL-6, and TNF-α levels were measured using ELISA kits. (**G**) BV-2 microglial cells were treated with TAK-242 (1μM), C29 (100μM), or serum-free medium for 60min, followed by treatment with heat-killed bacteria (MOI of 100) or serum-free medium for 6h and western blotting with anti-p-NF-κB p65, anti-NF-κB p65, and anti-GAPDH antibodies. Data from three independent experiments are presented as mean ± SD; Student's *t*-test, **$p<0.01$ and ***$p<0.001$ compared the *P. gingivalis*-LPS group with the *E. coli*-LPS group; one-way ANOVA, *$p<0.05$, **$p<0.01$, and ***$p<0.001$ compared to the control group, the heat-killed bacteria groups or the heat-killed bacteria plus TAK-242 groups.

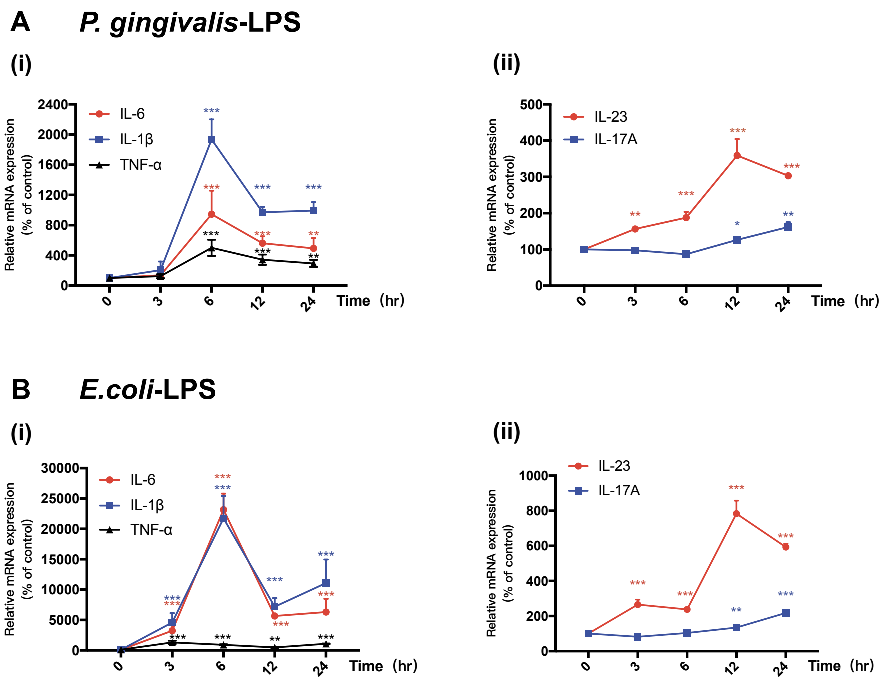

Figure S4. Inflammatory cytokine gene expression in *P. gingivalis*-LPS Standard and *E. coli*-LPS Standard stimulated BV-2 microglial cells. BV-2 microglial cells were treated with 1μg/ml Standard-LPS for 0-24h, and RT-PCR was performed; two-way ANOVA, *$p<0.05$, **$p<0.01$, and ***$p<0.001$ compared to the 0hr group. Data from three independent experiments are presented as mean ± SD.

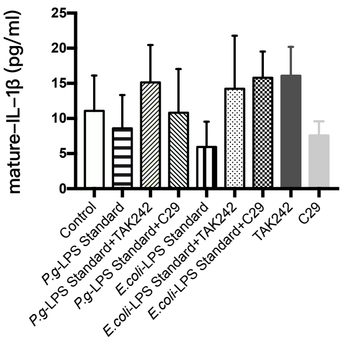

Figure S5. Mature-IL-1β release in *P. gingivalis*-LPS Standard and *E. coli*-LPS Standard stimulated BV-2 microglial cells. BV-2 microglial cells were treated with TAK-242 (1μM), C29 (100μM) or serum-free medium for 60min, following by treatment with 1μg/ml Standard-LPS or serum-free medium for 6h, and mature-IL-1β level were measured using ELISA kits. Data from three independent experiments are presented as mean ± SD.
